# The immediate and delayed effects of group activities on Chinese college students’ empathy: a longitudinal tracking study

**DOI:** 10.3389/fpsyg.2025.1505333

**Published:** 2025-05-27

**Authors:** Chuang Xu, Wenting Gong, Jian-Hong Ye, Fangyu Fu

**Affiliations:** ^1^School of Marxism, Hunan Institute of Technology, Hengyang, China; ^2^Faculty of Education, Beijing Normal University, Beijing, China; ^3^School of International Studies, Hunan Institute of Technology, Hengyang, China; ^4^National Institute of Vocational Education, Beijing Normal University, Beijing, China; ^5^School of Chemical and Environmental Engineering, Hunan Institute of Technology, Hengyang, China

**Keywords:** college students, group activities, empathy, immediate effects, delayed effects

## Abstract

Although the role of empathy in reducing campus bullying has been receiving increasing attention, empirical research on the development of empathy and its delayed effects among college students is lacking. To examine the immediate and delayed effects of group activities on increases in empathy among college students, this study randomly assigned 90 first-year students from a Chinese university into control, intervention, and delayed intervention groups. The groups with interventions participated in multiple sessions of counselling activities, and the Interpersonal Reactivity Index-C scale was used as a pre-test, post-test, and re-test measure to assess empathy levels in each group. Additionally, interview transcripts were employed to verify the effectiveness of the study. The results showed that group activities were significantly effective in enhancing the level of empathy among college students. Furthermore, the effect of group activities in enhancing empathy levels among college students tended to diminish over time but was maintained at a relatively high level for a certain period. This finding reveals that the development of empathy requires ongoing attention and support. Future studies could further explore the effects of different types of interventions on empathy levels, and the durability and stability of delayed intervention effects can be investigated in long-term follow-up studies. These findings will contribute to the development of more effective strategies for preventing and intervening in campus bullying, thereby providing theoretical and practical support for more harmonious campus environments.

## 1 Introduction

In recent years, an increasing number of instances of school bullying have come to light with the dissemination of information over the Internet (Cretu and Morandau, 2022; Saleem et al., 2021). Despite a series of measures at the national level to address the issue of school bullying, this societal ailment persists, presenting new trends and challenges. Furthermore, with the development of social media and the Internet, school bullying is no longer confined to traditional face-to-face forms but has rapidly spread within virtual spaces, presenting new paradigms (Abaido, 2020; Ye et al., 2024). This makes the addressing and handling school bullying more complex, requiring more comprehensive and innovative approaches (Swearer et al., 2009). From a social psychological perspective, school bullying is closely tied to students’ mental states, with empathy deficits or insufficiency often playing a key role (Hawley and Williford, 2015; Casey et al., 2017). Mental health education is a school-based anti-bullying strategy integrated into campus education, aiming to foster students’ healthy perspectives on interpersonal relationships and personal mental development (Wu, 2015). The ‘Notice on Strengthening the Management of Students’ Mental Health’ issued by the Ministry of Education, China (2021) explicitly requires universities to offer no less than 32 class hours of compulsory mental health education for undergraduates as a measure to comprehensively enhance students’ mental well-being.

Empathy is recognized as a crucial factor that influences the occurrence of bullying (Ye et al., 2024; Utomo, 2022). Studies have shown a negative correlation between levels of empathy and bullying: individuals with higher empathy tend to bully less (Lee et al., 2021; Liu et al., 2020).The importance of empathy lies not only in its direct impact on bullying behavior but also in its role as the cornerstone of good interpersonal communication (Rigby, 2007). Main et al. (2017) have stated that empathy is one of the most important conditions for interpersonal communication, as it enables individuals to accurately perceive the inner world of others as they do their own, never losing the condition of ‘as’ (Gallo, 1994; Zahavi, 2014). This psychological quality of empathy builds a deep sensory connection between individuals, providing a solid foundation for interpersonal communication (Howe, 2012). At the same time, victims of bullying generally exhibit lower-than-average levels of interpersonal communication skills (de Sousa et al., 2021). This may be due to the gradually developing desensitization to others’ emotions as a result of prolonged attacks and exclusion, or it could be attributed to heavy psychological burdens that make it difficult for them to express themselves effectively (Sung et al., 2018). This further emphasizes the importance of empathy, as it not only plays a role in preventing bullying but is also key in helping the victims of bullying to better adapt and respond to their adversities. However, the phenomenon of ‘empathy deficit’ is common in colleges and universities, indicating a noticeable inadequacy among college students in perceiving others’ emotions and engaging in perspective-taking (Zhou, 2022). Adolescents with lower levels of empathy are more aggressive than their peers (Lovett and Sheffield, 2007). It is evident that the lack of empathy among college students is a major cause of the frequent occurrence of school bullying, both online and offline. Therefore, enhancing the level of empathy among college students has become an urgent task. By strengthening the development of empathy, their interpersonal communication skills can be effectively improved and the occurrence of aggressive behavior reduced, thereby mitigating instances of school bullying (Liu et al., 2020). Achieving this goal is crucial for promoting harmonious interactions among students, fostering a nurturing campus environment, and cultivating high-quality talent (Astor and Benbenishty, 2018).

Group activities, also known as group counseling, are a common method for empathy training, the value of which is acknowledged by scholars (Lim, 2019; Lu et al., 2020; Nurhasanah et al., 2019). Group counselling, originated in the United States and flourished during World War II (Leddick, 2011), was introduced to China in the early 1990s (Chen, 1995). Over time, the implementation of group activities as an intervention for interpersonal communication issues among college students has become a primary form of psychological counselling in colleges (Bao and Zhu, 1997). In Chinese higher education contexts, it is usually integrated with the mental health education courses primarily targeted at first-year students. Due to limitations in total class hours and space requirements, the group typically consists of no more than 30 individuals, with each session lasting 2–4 h. Previous research has reported that group activities have a significant positive impact on interpersonal communication issues among college students (Alden and Regambal, 2010). Empathy is considered a basis for effective response skills in interpersonal communication, serving as one of the fundamental principles of effective interpersonal communication (Rigby, 2007; Main et al., 2017; Çelik and Alpan, 2023). Some empirical findings have also demonstrated the significant effect of group counselling in enhancing participants’ empathy (Gunawan et al., 2019). In other words, through various methods and techniques, group activities can significantly enhance college students’ empathy levels. However, research that assesses the development of empathy and its delayed effects among college students is scarce (Bas-Sarmiento et al., 2019), and recent studies have suggested that empathy levels may increase, decrease, or remain stable over time (Bhatia and Shetty, 2023; Piumatti et al., 2020). Thus, the delayed effects of empathy training among college students have become an issue of concern. Given the correlation between empathy level enhancement and group activities, researchers believe it is crucial to assess their immediate and delayed effects on empathy levels, especially considering the frequent bullying incidents that have been highlighted (Bas-Sarmiento et al., 2019; Hamuddin et al., 2023).

This study aimed to cultivate empathy among college students through group activities and assess their immediate and delayed effects. The results of this study would help colleges and universities address the problem of covert school bullying more effectively and provide empirical support for the development of relevant policies. Accordingly, the research questions are as follows:

*RQ1*: How to design and implement effective empathy-training group activities without increasing students’ academic burden?

*RQ2*: Do group activities have a significant effect on the development of empathy among college students?

*RQ3*: What are the delayed effects on empathy levels among college students after participating in group activities?

## 2 Theoretical rationale

### 2.1 Activity theory

Activity theory is a theoretical framework for understanding how humans engage in purposeful, communicative collective activities (Foot, 2016). This theory emphasizes that human learning occurs through activities, whether they are individual actions or the outcomes of group interactions (Roschelle, 1995). Activities in specific settings typically involve simultaneous affective and cognitive processes, which can subtly influence empathy, one of the key social functions (Maliske et al., 2023). It goes beyond single activities, focusing on collective ones as analysis units. Engeström presented the third-generation activity theory with six components: subject, object, community, tools, rules, and division of labor (Engeström, 2001). It emphasizes multiple voices of subjects, objects, and communities in the system, better revealing complex, dynamic interactions in real-world activity systems. It also views activities as the source of cognitive development, holding that human cognition, shaped by social and cultural activities, are formed through interactions among tools, rules, and communities. Thus, activity theory highly aligns with group counseling in aspects like contextuality, sociality, and dynamics. In group activities, participants can explore and experience different situations and roles through structured activities and interactions, thereby cultivating their cognition as well as emotions, including a deeper sense of empathy (Alves-Oliveira et al., 2019). Activity theory also suggests that, through participation in group activities, not only do individuals acquire knowledge and skills but their attitudes and values are shaped, which is essential for the development of empathy (Mattelmäki et al., 2014). The structure and process of group activities provide college students with rich situations and experiences that stimulate emotional resonance and understanding, which deepens their awareness of others’ emotions and needs, thereby raising their empathy levels (Barbezat and Bush, 2013). Therefore, activity theory offers a theoretical framework and guidance for designing group activities to enhance college students’ empathy by identifying the key elements of group counseling, providing tools relevant to the objectives, and motivating participants’ agency.

### 2.2 Experiential learning theory

Experiential learning theory states that in group activities, individuals acquire knowledge and skills through direct participation and firsthand experience, leading to a deeper understanding and internalization of what has been learned (Akella, 2010). The Kolb Learning Cycle is central to experiential learning theory and consists of four stages: concrete experience, reflective observation, abstract conceptualization, and active experimentation (Lai et al., 2024). This theory emphasizes that, through practical engagement and interaction, individuals are encouraged to actively participate, identify issues, seek solutions, and continually adjust and develop their cognitive structure within group contexts. This theory aligns well with group counseling for several reasons. First, group counseling is inherently experiential, offering a structured platform for interaction, practice, reflection, and change (Kolb et al., 2014). Within group activities, role-playing and member interactions enable individuals to share experiences, perspectives, and emotions, thereby broadening their horizons and gaining a more comprehensive understanding of themselves and others (Passarelli and Kolb, 2011). Next, group counseling offers participants a chance for reflective observation. By drawing on personal experiences and engaging in reflection, they can gain a deeper understanding of others’ emotions and needs, which helps cultivate a richer and more authentic sense of empathy. In addition, group activity leaders guide participants in extracting abstract concepts, such as “What is empathy?” and “How to maintain empathy?” from their experiences. This process enhances participants’ problem-solving abilities and emotional expression. Finally, group counseling promotes the application of acquired knowledge and skills in novel situations (Salimon, 2022), which means students are active agents and what they have learned can exert influence on them even when they are no longer in the counseling contexts. This is the foundation for this study’s exploration of the delayed effects of empathy in college students.

### 2.3 Solution-focused brief therapy

Solution-focused brief therapy (SFBT), a psychotherapeutic approach emphasizing students’ strengths and resources to foster positive change, focuses on present and future goals rather than past problems (Bannink, 2007). The core assumption of SFBT is that individuals possess the ability to address their own issues, with emphasis on short-term effectiveness and individual initiative. It was widely used in schools to address issues like academic stress, interpersonal relationships, and cyberbullying (Dartina et al., 2024). For instance, SFBT-based counseling can reduce cyberbullying by encouraging students to focus on positive interactions and improving their coping skills (Wang et al., 2024). Its core techniques include sharing personal experiences, seeking exceptions to problems, identifying strengths or solutions, using miracle questions, giving compliments, and future-focusing (De Shazer et al., 2021). While past studies have shown SFBT’s positive effects in individual psychotherapy (Kim, 2008), it has been less applied in group counseling. Group activities are a form of group-based psychological counseling. Through interpersonal interactions within a group, individuals can gain self-awareness and acceptance via observation, learning, and experience. They can also adjust and improve their interpersonal relationships and learn new attitudes and behaviors, thereby fostering adaptive helping processes (Fan, 2005). This study integrated SFBT philosophy and techniques into group activity design, aiming to uncovering and reinforcing individuals’ positive traits. We concentrated on enhancing empathy, exploring their meaning in life, and cultivating mindfulness, rather than mere analysis and correction of students’ empathy deficits. In our group activity program design, we first break down big goals into small, actionable steps. This allows participants to start small and gradually work toward the larger objectives within a short period. Also, we encourage members to share their own resources, strengths, and skills. We emphasize the significance of positive feedback and support members in actively affirming and praising each other’s efforts and progress. We guide members to reflect and summarize, to share their experiences and gains, and to explore how to apply what they have learned to real-life situations. Furthermore, in this group activity, the teacher assumes a guiding and reinforcing role that is closely aligned with the philosophy of SFBT.

## 3 Materials and methods

### 3.1 Design of the group activity

Based on activity theory, experiential learning theory, and solution-focused brief therapy, and after consulting with a university mental health education center, we finalized a group counseling strategy comprising six sub-activities to enhance college students’ empathy. The group activity strategy also focuses on enhancing students’ meaning in life and mindfulness. However, we do not distinguish between them specially, as studies show mindfulness helps individuals better recognize their own and others’ values, thereby strengthening empathy, and meaning in life may play a mediating role between mindfulness and empathy (Garg, 2023). Here are the six sub-activities (see Table 1).

**Table 1:** ‘All the way with you’ program of psychological group activity.

Procedure	Sub-activities	Purpose of the activity	Content of the activity
1	Icebreaker games (15 min)	The building of a small team environment: Breaking down barriers, getting to know each other, and establishing team interaction	Teams were formed freely, with members of each team lining up at random, and the person at the front introduced themselves to the person behind them.
2	Emotional waves (20 min)	Development of interpersonal skills: (1) Examining students’ ability to express and recognize emotions in interpersonal communication (2) Developing interpersonal understanding and collaborative skills	Each group lined up vertically, and the last student in each team drew a note, the message of which could only be conveyed to the person in front using body language and emotions, until it reached the front of the line, where the message was spoken out loud.
3	Work in unison (25 min)	Teamwork skills development: (1) Developing teamwork and communication skills (2) Developing the responsibility and self-awareness of group members	Using numbers to represent actions: 1 represented a step forward, 2 a step backward, 3 a step to the left, and 4 a step to the right. The facilitator called out a number, to which each group must react correctly in the shortest time possible.
4	Life’s five acts (30 min)	The construct of empathy: Understanding the challenges of being ‘human’, enriching students’ imagination, while establishing a sensory connection between individuals.	Participants of the same kind played mutual guessing. The winner moved up and the loser stayed, starting from ‘egg’ (squatting with knees hugged) to ‘chick’ (half squat with arms extended downwards) to ‘bird’ (standing with arms extended sideways) to ‘monkey’ (standing with arms raised in front) to ‘man’ (exiting and sitting down). If failing at ‘man’, the participant had to start over from ‘egg’.
5	It’s not easy for you either (30 min)	Development of perspective-taking skills: Guiding students to feel and understand others through external factors such as movement, expression, and voice, fostering the psychological qualities of empathy and perspective-taking.	Within each group, participants were divided into two halves. The left half performed simple yoga poses, holding still for 1 min; the right half performed challenging yoga poses, holding briefly in position. The participants in each half then switched positions.
6	Bombardment of merits (30 min)	Temporarily setting aside one’s own subjective frame of reference, attempt to discover others’ strengths from their perspectives.	Take turns to have one group member sit in the center of the group, while the other members take turns to express and appreciate their positive traits.

Icebreaker: Self-introduction helps participants get a preliminary understanding of each other and lays the groundwork for in-depth interaction later on.

Emotional waves: Non-verbal communication plays an important role in emotional expression (Nguyen et al., 2024). This activity, focusing on body language and emotion transmission, aims to enhance students’ sensitivity and understanding of others’ emotions.

Work in unison: Teamwork requires effective communication and coordination (Dai et al., 2025). This activity, emphasizing quick responses and teamwork, aims to strengthen the rapport among team members and enhance individual responsibility within the team.

Life’s five acts: Role-playing and simulating life stages can help students better understand the challenges and emotions of different life stages (Gilbert et al., 2023).

It is not easy for you either: By experiencing yoga poses of varying difficulties, students can intuitively sense each other’s differences and challenges, promoting empathy through perspective-taking.

Bombardment of merits: Positive feedback and affirmation can enhance self-worth and promote positive relationship development (Weng et al., 2024). By identifying and praising others’ strengths, students can learn to appreciate others.

The first three sub-activities aimed to fully mobilize the initiative of the individual and meet their existential needs through the development of expressive, communicative, and co-operative skills, thus enhancing adaptability. ‘Life’s five acts’ and ‘It’s not easy for you either’, on the other hand, guided students to perceive and understand others through external factors such as movements, facial expressions, and voices via mutual practice and influence among members, thus constructing and strengthening students’ empathy. In the sub-activity of ‘Bombardment of merits’, we guided students to think about life and raise their awareness of the meaning of life through mutual encouragement among members, aiming to stimulate students’ confidence and positive mindset. Through the above group activity program, university students’ empathy is expected to be boosted in multiple dimensions, and their interpersonal and teamwork skills are also expected to be enhanced (Muhammad and Muhammad, 2024).

### 3.2 Procedures and participants

This study recruited 90 undergraduate students (51 male, 39 female) from three classes at a technological college in Hunan Province, China, during the first semester of the 2023 academic year. Among them, 37 were only-children. All participants were freshmen aged 18 or older. After the Academic Ethics Review Committee of Hunan Institute of Technology approved the study, participants learned about its objective during a class meeting in October and signed informed consent forms. The researchers guaranteed that the study data would be used solely for the purpose of this study and that the privacy of each participant would be safeguarded. During the study, participants were assured of their right to withdraw their research data at any stage. The 90 college students were randomly divided into three groups, with each group 30 students. Group A served as the control group, receiving no intervention. Group B underwent timely intervention, engaging in a 150-min group activity program. Group C received delayed intervention, with the group activity scheduled 1 month after that of Group B, to analyze differences caused by the scheduling variance. In this study, the group activity called ‘All the way with you’ was conducted in the same growth counselling room. To avoid disparities in research outcomes due to varying styles and abilities of different teachers, the experiments for Groups B and C were supervised by the same postgraduate student in psychology and a teacher with a doctorate in education. The introduction and background music of each sub-activity were debugged and rehearsed before the group activity.

### 3.3 Study tools

The Interpersonal Reactivity Index (IRI), a tool developed to measure empathy, is widely recognized by scholars for its high reliability and validity (Yang and Kang, 2020). The Chinese version of the Interpersonal Reactivity Index-C (IRI-C), translated by Siu and Shek (Siu and Shek, 2005), was used as the measurement in this study. The scale consisted of 22 items across three dimensions: Personal Distress, Fantasy Scale, and Empathy Scale. Because of its proven reliability and validity among Chinese college students, it was deemed suitable for use in this study. The IRI-C is scored on a 5-point Likert scale, ranging from 1, not appropriate, to 5, very appropriate. The internal consistency coefficient for all questionnaire data was 0.816, with a cumulative explained variance of 63.686%. The skewness ranged from 0.021 to 0.785 and the kurtosis from 0.140 to 1.670, indicating that the scale possessed good reliability and validity and could be used as an empathy assessment tool in the Chinese population. The IRI-C scale completion timeline was as follows: the pre-tests for Groups A/B/C were all completed during the week of 17 September 2023; the post-tests for Groups A and B were completed on 8 October 2023; the post-tests for Group C were completed on 5 November 2023 to assess the immediate effects of the group activities; and the re-tests for Groups A, B and C were completed on 14 January 2024 to assess the delayed effects of the group activities. Data were collected using Wenjuanxing, a Chinese online survey platform. A total of 270 questionnaires were distributed, and all 270 responses received were valid.

The post-group-activity interviews were conducted in a manner that allowed participants to freely express their experiences and viewpoints. Two students from Group B and one student from Group B were randomly invited to come up to the stage to share their feelings after the group counseling session. Each student spoke for about 5 min. Their speeches were recorded, transcribed, and labeled as S1, S2, and S3. Several guided questions for students to talk were given by the two supervisors in the group activity: What did you do before participating in the activity? What were you thinking during the activity? and How did you feel after the activity? Using random invitations to speak, we ensured objectivity and comprehensiveness of the feedback, allowing researchers to gain a broader understanding of students’ experiences and changes.

### 3.4 Data analysis

SPSS version 26.0 was used for statistical analysis. The sample was summarized using descriptive statistics to clarify the current state of empathy among college students, including the total score mean and standard deviation. Additionally, ANOVA was employed to examine the differences among Groups A, B, and C, as well as the differences between the pre-test and post-test and between the post-test and re-test within the groups. The recorded interview materials, transcribed verbatim, were returned to the participants for verification to assess the effectiveness of the group activities on the enhancement of empathy levels.

## 4 Study results and discussion

### 4.1 Differences between groups

The pre-test data indicated that the empathy levels in each group were moderate to low, with no significant differences between the groups (*F* = 1.817, *p* = 0.169). This was consistent with the findings of Saleem et al. (2021); Lee et al. (2021); and Liu et al. (2020), suggesting that empathy levels among first-year students needed improvement. Post-test results showed that empathy levels were significantly higher in Groups B and C, which received the intervention, than in the non-intervened Group A (*F* = 29.745, *p* < 0.001), and all 60 participants involved in the group activities expressed satisfaction with the quality of the activities, stating that the atmosphere of the group activities was excellent and had a significant impact on them. Feedback such as ‘I am more caring than before’, ‘I am more active and bolder than before’, and ‘I am more understanding of others’ challenges than before’ further illustrated the significant effect of the group activities in enhancing empathy levels among college students. This was consistent with the findings of Lim (2019); Lu et al. (2020); and Nurhasanah et al. (2019). The re-test results, conducted 2–3 months later, were consistent with the post-test results. The empathy scores for Groups B and C were significantly higher than those for Group A, as shown in Table 2.

**Table 2:** Analysis of differences among groups.

Groups	Pre-test (*M* ± *SD*)	Post-test (*M* ± *SD*)	Re-test (*M* ± *SD*)	*F*	*Post-hoc* tests
Group A	2.631 ± 0.403	2.633 ± 0.430	2.787 ± 0.489	1.227	–
Group B	2.860 ± 0.523	3.506 ± 0.505	3.187 ± 0.578	10.848***	Post-test > Pre-test
Group C	2.787 ± 0.489	3.312 ± 0.440	3.304 ± 0.444	12.872***	Post-test > pre-test; re-test > pre-test
*F*	1.817	29.745***	8.564***		
*Post-hoc* tests	–	B > A; C > A	B > A; C > A		

***p < 0.001.

### 4.2 Differences within groups

The pre-test, post-test, and re-test results for Group A showed no significant differences (*F* = 1.227, *p* = 0.298). However, the mean scores of empathy levels showed an increasing trend, indicating that the empathy levels of first-year students improved gradually after a semester of study and life experience. The data for Group B showed that the immediate effect of the group activities on enhancing empathy levels was significant (*F* = 10.848, *p* < 0.001), with the mean score increasing from 2.860 to 3.506. However, it is worth noting that the mean score on the re-test, conducted 3 months later, had decreased to 3.187. Although there was an increase compared with the pre-test mean score of 2.860, the difference was not significant, indicating that the effect of the group activities on empathy levels among college students diminished over time. The data from Group C also supported this, as the mean score on the re-test, conducted 2 months later, had decreased by 0.008 from the post-test mean score. However, unlike Group B, the re-test scores remained significantly higher than the pre-test scores, indicating that the effect of the group activities on enhancing empathy levels among college students was maintained at a relatively high level for a substantial period of time and did not disappear, as shown in Table 2.

### 4.3 Immediate effects of group activities on empathy among college students

The results of the study showed that the level of empathy among college students increased significantly after they participated in group activities. Consistent with the findings of Lim (2019); Lu et al. (2020); and Nurhasanah et al. (2019), group activities can be considered an effective means of enhancing empathy levels. Empathy has been considered a crucial element in interpersonal communication (Rigby, 2007; Main et al., 2017; Howe, 2012; Çelik and Alpan, 2023), validating the efficacy of group activities as an effective means of enhancing interpersonal communication. Consistent with the findings of Alden and Regambal (2010), group activities have favorable effects in improving interpersonal interactions among college students (Bao and Zhu, 1997). A series of counselling activities have sparked college students’ ability to empathize, enabling them to further understand life’s challenges and enhancing their interpersonal communication skills through increased self-awareness and understanding of others. This kind of training not only helps reduce the phenomenon of “empathy deficit” but also improves college students’ ability to empathize and understand others’ emotions, thereby reducing aggressive behaviors on campus and fostering a more positive atmosphere (Lee et al., 2021; Liu et al., 2020). In addition, within the designed group activity setting, empathy is significantly enhanced through the reshaping of emotions and cognition, further confirming activity theory (Roschelle, 1995; Maliske et al., 2023). The findings of this study provide empirical support for the effectiveness of group activities in enhancing empathy among college students, emphasizing their positive role in improving interpersonal relationships and fostering positive affective attitudes. It also offers implicit theoretical guidance and can serve as a practical reference for colleges and universities to adopt empathy-training group activities as a low-cost and effective measure for bullying prevention and control.

### 4.4 Delayed effects of group activities on empathy among college students

The results of the study showed that the level of empathy among college students decreased within 2–3 months after the group activities. This finding was consistent with those of Bhatia and Shetty (2023), that the effectiveness of group activities in enhancing empathy among college students diminished over a semester, which was an unavoidable trend (Piumatti et al., 2020). This may be explained by the group atmosphere being more conducive to stimulating self-expression among students, leading to fluctuations in empathy levels over a certain period. It is worth noting that, although levels of empathy declined after 2–3 months, they were still higher than before the group activities. This indicates that group activities remain effective in enhancing empathy among university students over a certain period (Bas-Sarmiento et al., 2019; Hamuddin et al., 2023). This also demonstrates the ubiquity and sustainability of learning within experiential learning theory, and provides new perspectives for further deepening the application of experiential learning theory in group activities (Kolb et al., 2014; Salimon, 2022). However, because of the limitations of this study, more in-depth research and investigation are needed to further explore the mechanism of delayed effects.

The results of the study remind us that, although group activities have a significant effect on empathy levels in the short term, there may be a declining trend in the long term. Hence, to better maintain the improvement in empathy levels, it may be necessary to design ongoing training sessions or reminder mechanisms following group activities to help students consolidate and apply the empathy skills they have learned (Bhatia and Shetty, 2023; Piumatti et al., 2020). This also contributes to a more comprehensive understanding of the long-term impact and sustainability of group activities in fostering empathy among college students.

### 4.5 Immediate feedback after SFBT group activities

Group activities effectively create a positive team atmosphere and promote communication and cooperation among members, in line with prior studies (Dai et al., 2025). In a safe and vibrant environment, students are more likely to open up, engage actively, and develop strong interpersonal communication and emotional expression (Bao and Zhu, 1997). As one student noted, *“This activity was magical. I would be very nervous to speak in a crowd and would turn red and feel helpless. But after interacting with the group, everyone encouraged and comforted me, and gradually, I could even hear my own laughter. I completely let go of my inhibitions!”(S1).*

Ample interaction and mutual assistance enable a more comprehensive and objective self-perception. In an environment of mutual acceptance and appreciation, individuals feel more comfortable revealing their true selves and expressing emotions (Nguyen et al., 2024). This fosters deep trust among team members, making it easier to share thoughts and feelings (Weng et al., 2024). As one student recorded, “*Initially, I never expected myself to open up so much. After all, everyone had just started college and wasn’t very familiar with each other. Who would share their real experiences and thoughts? But unexpectedly, after completing the mutual praise task in the “Bombardment of merits” sub-activity, our group continued chatting with enthusiasm. I even shared details about how I interacted with my peers. This transformation was beyond my own expectations!”(S3)* These echo the importance of team atmosphere in virtual teams, highlighting dimensions like team performance, communication, and cohesion (Muhammad and Muhammad, 2024).

Students also reported a more profound understanding of others’ situations, reflecting the positive effects of empathy training. This enhances their emotional intelligence and understanding (Muhammad and Muhammad, 2024; Del Barrio et al., 2024). For instance, S2 shared her reflective feedback, noting that everyone faces challenges and that empathizing requires effort. *“At first, I hesitated and felt nervous when I saw a classmate squatting for a long time, looking at me with pleading eyes. Later, when I returned to being the “egg,” he came over to help me without hesitation. I was really touched!”(S2).* This indicates that experiential learning can strengthen students’ empathy (Dai et al., 2025). When facing team-task difficulties, mutual support and collaboration are essential. These shared experiences highlight the importance of interdependence within a team, fostering greater respect and understanding among members (Gilbert et al., 2023). A positive team environment cultivates students’ emotional intelligence, making them better at expressing, listening to, and comprehending others’ emotional needs (Humphrey et al., 2007). The process of emotional resonance and mutual understanding shapes more empathetic college students.

## 5 Conclusion and implications

The significant effects of group activities in enhancing empathy levels among college students have been highlighted. Through systematic group activity planning and implementation, supported by quantitative and qualitative data and the thorough validation and organization of theoretical frameworks, this study has revealed the practical impact of group activities on empathy levels among college students. The results showed that group activities are significantly effective in enhancing the level of empathy among college students. Furthermore, the effect of group activities on enhancing empathy levels among college students tends to diminish over time but is maintained at a relatively high level for a certain period. The findings are favorable for educational administrators to formulate targeted empathy development programs, which holds significant importance in promoting the comprehensive emotional development of students (Yu et al., 2021). At the same time, due to the current limitations on class hours for mental health education, the value of the SFBT—integrated group activity program in promotion has also been highlighted (Dartina et al., 2024; Wang et al., 2024; Wang et al., 2024).

This study innovatively advances existing literature in some aspects. Firstly, in terms of group intervention paradigms, prior studies have confirmed the empathy-enhancing effect of psychological group activities. However, they mainly focus on long-term interventions (over 8 weeks) without structured operation guidelines (Yu et al., 2021). This study develops a standardized, solution-focused brief therapy group counseling approach. Unlike the minimum 8-week intervention proposed by Del Barrio et al. (2024), it confirms that six well-designed sub-activities modules can also significantly boost empathy within limited class hours. This provides universities with an empirical solution to the challenge of mental health education time constraints.

Secondly, the delayed effects of empathy training among college students have been explored in depth. In addition to immediate effects as explored in literature (Yu et al., 2021), this study also conducted continuous follow-up and evaluation of the training effectiveness. Through a longitudinal tracking design, this study found that although empathy levels showed some natural decay after 3 months, the post-test and re-test data remained significantly above the pre-test baseline (Salimon, 2022). This perspective helps to understand the long-term impact of empathy training, reveals that the cognitive restructuring from group activities has a lasting effect beyond emotional arousal. Meanwhile, by comparing immediate and delayed effects, a more comprehensive assessment of the effectiveness and sustainability of group activities could be made, providing valuable insights for future empathy training initiatives.

Our study also has significant implications for schools, students, families, and other relevant domains. In addressing the current issue of ‘empathy deficit’ among college students, institutions should optimize group activity strategies to boost their effectiveness in fostering empathy. This involves creating more inclusive and stimulating activity settings, offering diverse participation opportunities, and incorporating advanced training techniques and methods. It is imperative to broaden the limited scope of group activities so that a larger number of students can benefit from them. In addition, universities must integrate empathy education into their curricula (Muhammad and Muhammad, 2024; Del Barrio et al., 2024; Yu et al., 2021). They should strengthen general mental health education and guide students in comprehending the concept, significance, and application of empathy in interpersonal relationships. This will help cultivate students with sound psychological qualities and promote holistic student development (Wu, 2015). At the student level, there should be an emphasis on promoting peer interaction, encouraging students to establish more open and trusting relationships with their peers, and enhancing emotional connections through communication and sharing of feelings. Students are also encouraged to share their feelings and experiences with each other to promote empathy and resonance. Families and society should also work together to provide a more supportive and understanding environment for college students. Through these comprehensive efforts, it is expected that a more empathetic college student population will be fostered, thus reducing the occurrence of campus bullying and building a more harmonious, friendly, and mutually respectful society.

## 6 Limitations of the study and future recommendations

This study had certain limitations. First, although we conducted a longitudinal study within a semester, tracking across semesters might introduce uncertainties affecting empathy’s delayed effects. Second, while SFBT empathy—training group activities can be replicated and promoted, real—world educational settings have many interfering factors. For instance, most mental health education courses may lack sufficient time for all students participating in 2–4 h group activities. Additionally, the mechanism by which students’ empathy growth alleviates school bullying behaviors has not been sufficiently verified, it has only been partially explained through literature.

To comprehensively understand the long-term effects of empathy training, future research could employ sophisticated tracking and multi—validation strategies. Divide tracking into monthly or quarterly units, conduct interventions and data collection across long vacations, analyze environmental moderators via Structural Equation Modeling to spot key fluctuation points in delayed empathy effects, and support enhanced intervention cycles. To overcome course hour limits, future research could explore simplified intervention modules. For example, design “10-min embedded classroom activities’ based on microlearning theory, or develop mobile app scenario tasks. And then test these fragmented interventions’ effectiveness on empathy maintenance through randomized controlled trials. Finally, the mechanism linking empathy to covert bullying can be tested empirically with multimodal data to show how empathy growth curbs such bullying. Future research could employ quasi—experiments to compare the behavior of intervention and control groups in simulated campus conflicts, such as the frequency of verbal attacks or supportive behaviors. This would establish practical causal—chain evidence and overcome the current study’s limits.

## Data Availability

The raw data supporting the conclusions of this article will be made available by the authors, without undue reservation.

## References

[ref1] Abaido G. M. (2020). Cyberbullying on social media platforms among university students in the United Arab Emirates. Int. J. Adolesc. Youth 25, 407–420. doi: 10.1080/02673843.2019.1669059

[ref2] Akella D. (2010). Learning together: Kolb’s experiential theory and its application. J. Manag. Organ. 16, 100–112. doi: 10.5172/jmo.16.1.100

[ref3] Alden L. E. Regambal M. J. (2010). “Interpersonal processes in the anxiety disorders” in Handbook of interpersonal psychology: Theory, research, assessment, and therapeutic interventions. eds. Horowitz L. M. Strack S. (Hoboken: Wiley), 449–469.

[ref4] Alves-Oliveira P. Sequeira P. Melo F. S. Castellano G. Paiva A. (2019). Empathic robot for group learning: a field study. ACM Trans. Hum.-Robot Interact. 8, 1–34. doi: 10.1145/3300188

[ref5] Astor R. A. Benbenishty R. (2018). Bullying, school violence, and climate in evolving contexts: Culture, organization, and time. New York: Oxford University Press, 182–183.

[ref6] Bannink F. P. (2007). Solution-focused brief therapy. J. Contemp. Psychother. 37, 87–94. doi: 10.1007/s10879-006-9040-y

[ref7] Bao Y. Zhu F. (1997). “Intervention experiment research on college students’ interpersonal relationship by solution-focused group counseling” in Proceedings of the 2017 international conference on service systems and service management (ICSSSM), Dalian, China, 16–18 June 2017 (Piscataway: IEEE), 1–4. doi: 10.1109/ICSSSM.2017.7996234

[ref8] Barbezat D. P. Bush M. (2013). Contemplative practices in higher education: Powerful methods to transform teaching and learning. San Francisco: Jossey-Bass, 174–188.

[ref9] Bas-Sarmiento P. Fernández-Gutiérrez M. Díaz-Rodríguez M. Carnicer-Fuentes C. Castro-Yuste C. García-Cabanillas M. J.. (2019). Teaching empathy to nursing students: a randomised controlled trial. Nurse Educ. Today 80, 40–51. doi: 10.1016/j.nedt.2019.06.002, PMID: 31252353

[ref10] Bhatia G. Shetty J. V. (2023). Trends of change in empathy among Indian medical students: a two-year follow-up study. Indian J. Psychol. Med. 45, 162–167. doi: 10.1177/02537176221104688, PMID: 36925484 PMC10011843

[ref11] Casey E. A. Lindhorst T. Storer H. L. (2017). The situational-cognitive model of adolescent bystander behavior: modeling bystander decision-making in the context of bullying and teen dating violence. Psychol. Violence 7, 33–44. doi: 10.1037/vio0000033, PMID: 33884221 PMC8057727

[ref12] Çelik Ö. C. Alpan G. (2023). The impact of an effective communication course with enhanced student engagement on communication skills and empathic tendency of preservice teachers. Educ. Proc. Int. J. 12, 33–58. doi: 10.22521/edupij.2023.122.3

[ref13] Chen C. P. (1995). Group counseling in a different cultural context: several primary issues in dealing with Chinese clients. Group 19, 45–55.

[ref14] Cretu D. M. Morandau F. (2022). Bullying and cyberbullying: a bibliometric analysis of three decades of research in education. Educ. Rev. 76, 371–404. doi: 10.1080/00131911.2022.2034749

[ref15] Dai Z. Yang Y. Chen Z. Wang L. Zhao L. Zhu X.. (2025). The role of project-based learning with activity theory in teaching effectiveness: evidence from the internet of things course. Educ. Inf. Technol. 30, 4717–4749. doi: 10.1007/s10639-024-12965-9

[ref16] Dartina V. Nabila S. Alfaiz A. Maharani I. F. (2024). Systematic Literature Review: Penerapan Layanan Konseling Kelompok Solution Focused Brief Therapy (SFBT) pada Peserta Didik di Sekolah Menengah. Indones. J. Educ. Couns. 8, 36–46. doi: 10.30653/001.202481.319

[ref17] De Shazer S. Dolan Y. Korman H. Trepper T. McCollum E. Berg I. K. (2021). More than miracles: The state of the art of solution-focused brief therapy. New York: Routledge.

[ref18] de Sousa M. L. Peixoto M. M. Cruz S. F. (2021). The association between externalizing and internalizing problems with bullying engagement in adolescents: the mediating role of social skills. Int. J. Environ. Res. Public Health 18:10444. doi: 10.3390/ijerph181910444, PMID: 34639743 PMC8507938

[ref19] Del Barrio L. G. Rodríguez-Díez C. Gea A. Arbea L. Pereira J. Díez N. (2024). Impact of a longitudinal course on medical professionalism on the empathy of medical students. Patient Educ. Couns. 119:108042. doi: 10.1016/j.pec.2023.108042 37978022

[ref20] Engeström Y. (2001). Expansive learning at work: toward an activity theoretical reconceptualization. J. Educ. Work. 14, 133–156. doi: 10.1080/13639080020028747

[ref21] Fan F. (2005). The development of group counseling in China: review and Prospect. J. Tsing Hua Univ. 6, 62–69. doi: 10.13613/j.cnki.qhdz.001093

[ref22] Foot K. A. (2016). Activity theory. In The International encyclopedia of communication theory and philosophy. eds. Bruhn Jensen K. Craig R. T. Pooley J. Rothenbuhler E. W. (Wiley), 1–8. doi: 10.1002/9781118766804.wbiect033

[ref23] Gallo D. (1994). “Educating for empathy, reason, and imagination” in Re-thinking reason: New perspectives in critical thinking. ed. Walters K. S. (New York: State University of New York Press), 43–60.

[ref24] Garg L. (2023). A correlational study on psychological distress, mindfulness and meaning in life among young adults. Int. J. Interdiscip. Approaches Psychol. 1, 1–14. doi: 10.61113/ijiap.v1i1.60

[ref25] Gilbert K. L. Baker E. A. Bain K. Flood J. Wolbers J. (2023). Say something, do something: evaluating a forum theater production to activate youth violence prevention strategies in schools. Int. J. Environ. Res. Public Health 21:39. doi: 10.3390/ijerph21010039, PMID: 38248504 PMC10815014

[ref26] Gunawan I. M. S. Wibowo M. E. Purwanto E. Sunawan S. (2019). Group counseling of values clarification to increase middle school students’ empathy. Psicol. Educativa. Rev. Psicólogos Educ. 25, 169–174. doi: 10.5093/psed2019a5

[ref27] Hamuddin B. Rahman F. Pammu A. Baso Y. S. Derin T. (2023). Mitigating the effects of cyberbullying crime: a multi-faceted solution across disciplines. Int. J. Innov. Res. Sci. Stud. 6, 28–37. doi: 10.53894/ijirss.v6i1.1079

[ref28] Hawley P. H. Williford A. (2015). Articulating the theory of bullying intervention programs: views from social psychology, social work, and organizational science. J. Appl. Dev. Psychol. 37, 3–15. doi: 10.1016/j.appdev.2014.11.006

[ref29] Howe D. (2012). Empathy: What it is and why it matters. Hampshire: Palgrave Macmillan, 63–73.

[ref30] Humphrey N. Curran A. Morris E. Farrell P. Woods K. (2007). Emotional intelligence and education: a critical review. Educ. Psychol. 27, 235–254. doi: 10.1080/01443410601066735, PMID: 40101104

[ref31] Kim J. S. (2008). Examining the effectiveness of solution-focused brief therapy: a Meta-analysis. Res. Soc. Work. Pract. 18, 107–116. doi: 10.1177/1049731507307807

[ref32] Kolb D. A. Boyatzis R. E. Mainemelis C. (2014). “Experiential learning theory: previous research and new directions” in Perspectives on thinking, learning, and cognitive styles. eds. Sternberg R. J. Zhang L. F. (New York: Routledge), 227–247.

[ref33] Lai L. She L. Li C. (2024). Online teaching model in the context of blended learning environment: experiential learning and TAM. Educ. Inf. Technol. 29, 17235–17259. doi: 10.1007/s10639-024-12465-w

[ref34] Leddick G. R. (2011). “The history of group counseling” in The Oxford handbook of group counseling. ed. Conyne R. K. (New York: Oxford University Press), 52–60.

[ref35] Lee J. Cheung H. S. Chee G. Chai V. E. (2021). The moderating roles of empathy and attachment on the association between latent class typologies of bullying involvement and depressive and anxiety symptoms in Singapore. Sch. Ment. Heal. 13, 518–534. doi: 10.1007/s12310-021-09411-3

[ref36] Lim A. R. (2019). Study of differences in empathic ability and emotional intelligence according to college students’ counseling course-taking experience and major. J. Korea Acad. Ind. Coop. Soc. 20, 103–110. doi: 10.5762/KAIS.2019.20.9.103

[ref37] Liu C. Wang L. Liu Z. Li Y. Yuan G. (2020). Mindfulness and cyberbullying among Chinese adolescents: the mediating roles of perceived social support and empathy. Violence Vict. 35, 815–827. doi: 10.1891/VV-D-19-00104, PMID: 33372111

[ref38] Lovett B. J. Sheffield R. A. (2007). Affective empathy deficits in aggressive children and adolescents: a critical review. Clin. Psychol. Rev. 27, 1–13. doi: 10.1016/j.cpr.2006.03.003, PMID: 16697094

[ref39] Lu Y. Hill C. E. Hancock G. R. Keum B. T. (2020). The effectiveness of helping skills training for undergraduate students: changes in Ethnocultural empathy. J. Couns. Psychol. 67, 14–24. doi: 10.1037/cou0000404, PMID: 31697121

[ref40] Main A. Walle E. A. Kho C. Halpern J. (2017). The interpersonal functions of empathy: a relational perspective. Emot. Rev. 9, 358–366. doi: 10.1177/1754073916669440

[ref41] Maliske L. Z. Schurz M. Kanske P. (2023). Interactions within the social brain: co-activation and connectivity among networks enabling empathy and theory of mind. Neurosci. Biobehav. Rev. 147:105080. doi: 10.1016/j.neubiorev.2023.105080, PMID: 36764638

[ref42] Mattelmäki T. Vaajakallio K. Koskinen I. (2014). What happened to empathic design? Des. Issues 30, 67–77. doi: 10.1162/DESI_a_00249

[ref43] Muhammad S. A. Muhammad S. R. (2024). Empathy unlocked: enhancing emotional intelligence skills using communication workshop for undergraduates medical and dental students. JMMC 15, 54–61. doi: 10.62118/jmmc.v15i1.475

[ref44] Nguyen A. J. Hersh J. Beahm L. Henderson Smith L. Newman C. Birchfield K.. (2024). Coping power-rural: iterative adaptation of an evidence-based preventive intervention for rural upper elementary and middle schools. Sch. Ment. Heal. 16, 776–792. doi: 10.1007/s12310-024-09632-2

[ref45] Nurhasanah N. Neviyarni S. Effendi Z. M. (2019). The effectiveness of group counseling with role-playing techniques to increase student empathy. Int. J. Appl. Couns. Soc. Sci. 1, 54–61. doi: 10.24036/005304ijaccs

[ref46] Passarelli A. M. Kolb D. A. (2011). “The learning way: learning from experience as the path to lifelong learning and development” in The Oxford handbook of lifelong learning. ed. London M. (New York: Oxford University Press), 70–90.

[ref47] Piumatti G. Abbiati M. Baroffio A. Gerbase M. W. (2020). Empathy trajectories throughout medical school: relationships with personality and motives for studying medicine. Adv. Health Sci. Educ. 25, 1227–1242. doi: 10.1007/s10459-020-09965-y, PMID: 32095990

[ref48] Rigby K. (2007). Bullying in schools and what to do about it: Revised and updated. Camberwel: Acer Press, 206–212.

[ref49] Roschelle J. (1995). “The construction of shared knowledge in collaborative problem solving” in Computer supported collaborative learning. ed. O’Malley C. (Berlin: Springer. Germany), 69–99.

[ref50] Saleem S. Khan N. F. Zafar S. (2021). Prevalence of cyberbullying victimization among Pakistani youth. Technol. Soc. 65:101577. doi: 10.1016/j.techsoc.2021.101577

[ref51] Salimon F. (2022). Experiential learning. Int. J. Res. Publ. Rev. 3, 2363–2366. doi: 10.55248/gengpi.2022.31276

[ref52] Siu A. M. Shek D. T. (2005). Validation of the interpersonal reactivity index in a Chinese context. Res. Soc. Work. Pract. 15, 118–126. doi: 10.1177/1049731504270384

[ref53] Sung Y. H. Chen L. M. Yen C. F. Valcke M. (2018). Double trouble: the developmental process of school bully-victims. Child Youth Serv. Rev. 91, 279–288. doi: 10.1016/j.childyouth.2018.06.025

[ref54] Swearer S. M. Espelage D. L. Napolitano S. A. (2009). Bullying prevention and intervention: Realistic strategies for schools. New York: Guilford Press, 3–4.

[ref55] Utomo K. D. M. (2022). Investigations of cyber bullying and traditional bullying in adolescents on the roles of cognitive empathy, affective empathy, and age. Int. J. Instr. 15, 937–950. Retrieved from: https://e-iji.net/ats/index.php/pub/article/view/420

[ref56] Wang C. Y. Shen L. Shields J. Huang Q. C. Wu Y. J. Yin J. W.. (2024). The efficacy of an SFBT-based positive psychology intervention in promoting university students’ post-traumatic growth and psychological resilience after the COVID-19 pandemic: a quasi-experiment. Res. Soc. Work. Pract. doi: 10.1177/10497315241229667

[ref57] Weng C. Kassaw K. Tsai P. S. Lee T. J. (2024). Does scratch animation for sustainable development goals (SDGs) with AI-comics impact on student empathy, self-efficacy, scriptwriting, and animation skills? Educ. Inf. Technol. 29, 18097–18120. doi: 10.1007/s10639-024-12576-4

[ref58] Wu X. (2015). Analysis on the development of mental health education for university students in the new century. Cross-Cultural Communication 11, 80–84. doi: 10.3968/7002

[ref59] Yang H. Kang S. J. (2020). Exploring the Korean adolescent empathy using the interpersonal reactivity index (IRI). Asia Pac. Educ. Rev. 21, 339–349. doi: 10.1007/s12564-019-09621-0

[ref60] Ye J. H. Chen M. Y. Wu Y. F. (2024). The causes, counseling, and prevention strategies for maladaptive and deviant behaviors in schools. Behav. Sci. 14:118. doi: 10.3390/bs14020118, PMID: 38392471 PMC10885922

[ref61] Ye J. H. Yang X. Nong W. Wang M. Lee Y. S. (2024). Antecedents and outcomes of cyberbullying among Chinese university students: verification of a behavioral pathway model. Front. Public Health 12:1359828. doi: 10.3389/fpubh.2024.1359828, PMID: 38628849 PMC11019017

[ref62] Yu J. Parsons G. S. Lancastle D. Tonkin E. T. Ganesh S. (2021). “Walking in their shoes”: the effects of an immersive digital story intervention on empathy in nursing students. Nurs. Open 8, 2813–2823. doi: 10.1002/nop2.860, PMID: 33743185 PMC8363366

[ref63] Zahavi D. (2014). Self and other: Exploring subjectivity, empathy, and shame. New York: Oxford University Press, 99–110.

[ref64] Zhou Z. (2022). Empathy in education: a critical review. Int. J. Sch. Teach. Learn 16:2. doi: 10.20429/ijsotl.2022.160302

